# Disruption of the Functional Activity of Neutrophil Granulocytes as a Risk Factor for the Development of Lung Damage in Pregnant Women with COVID-19

**DOI:** 10.3390/cimb46020071

**Published:** 2024-01-25

**Authors:** Irina Anatolyevna Andrievskaya, Egor Mikhailovich Ustinov, Karen Sargisovich Lyazgian, Nataliya Alexandrovna Ishutina, Inna Victorovna Dovzhikova

**Affiliations:** Laboratory of Mechanisms of Etiopathogenesis and Recovery Processes of the Respiratory System at Non-Specific Lung Diseases, Far Eastern Scientific Center of Physiology and Pathology of Respiration, 22 Kalinina Str., Blagoveshchensk 675000, Russia; eustinov.asma@gmail.com (E.M.U.); lyazgyankaren@mail.ru (K.S.L.); ishutina-na@mail.ru (N.A.I.); dov_kova100@rambler.ru (I.V.D.)

**Keywords:** COVID-19, SARS-CoV-2, pneumonia, pregnancy, innate immunity, neutrophils, functional activity

## Abstract

Currently, the assessment of immune status in patients with COVID-19 is limited to determining the count of polymorphonuclear leukocytes and the phagocytic function of neutrophils, which is insufficient to understand the regulatory role of innate immunity cells in the development of pneumonia. However, no such studies have been conducted in pregnant women with COVID-19. The aim of this study was to investigate the functional state of neutrophil granulocytes in order to identify predictors of pneumonia severity risk in pregnant women with COVID-19. A clinical characterization of pregnant women with COVID-19 in addition to minimal and average lung changes was provided. The composition and ratio of morphological forms of leukocyte cells were studied. Cytochemical studies of neutrophil granulocytes were carried out and calculations of the mean cytological index (MCI) for succinate dehydrogenase, myeloperoxidase, and cationic proteins were performed. The number of NETs in blood smears was counted. Independent predictors of pneumonia severity in pregnant women with COVID-19 were calculated using regression analysis. The quality of the model was assessed using ROC analysis. In pregnant women with COVID-19 and an average volume of lung changes, the number of polymorphonuclear leukocytes (*p* = 0.03) and band neutrophils (*p* = 0.002) in the blood was significantly higher than in pregnant women with minimal lung changes. The MCI indicators of succinate dehydrogenase, cationic proteins, and myeloperoxidase in pregnant women with COVID-19 were reduced in relation to the control group (*p* < 0.0001). In blood smears of pregnant women with COVID-19 and an average volume of lung changes, the number of NETs increased (*p* = 0.002). Regression analysis showed that succinate dehydrogenase and NETs are independent predictors of pneumonia severity in pregnant women with COVID-19. Our study confirms the prognostic significance of low levels of neutrophilic succinate dehydrogenase and high levels of NETs in the blood of pregnant women with COVID-19. The combination of these two biomarkers is a significant reflection of the severity of pneumonia development in pregnant women with COVID-19. However, further research is needed to identify the mechanisms underlying this association.

## 1. Introduction

Neutrophilic granulocytes play a crucial role in the development of inflammation induced by COVID-19. Their involvement in lung damage and the pathogenesis of microvascular disorders has been established [1,2]. The increase in the circulation of neutrophilic granulocytes (neutrophilia) in COVID-19 patients may be considered as an adaptive mechanism aimed at virus neutralization through the formation of neutrophil extracellular traps (NETs) [3]. However, abnormal activation of neutrophils may act as a negative regulator of inflammation, leading to endothelial vessel damage, the subsequent platelet recruitment for extracellular vesicle release, and NET formation [4,5].

The elevated circulation of platelet-neutrophil complexes in COVID-19 patients may contribute to epithelial layer damage, alveolar fibrin deposition, and microthrombus formation [6]. According to Wu C. et al., the severity of lung involvement is associated with extensive neutrophil infiltration and high neutrophil counts in the blood of severe COVID-19 patients complicated by acute respiratory distress syndrome (ARDS) [7]. The correlation between neutrophilia, the development of ARDS, and fatal outcomes in COVID-19 has been demonstrated. Conversely, the development of neutropenia, associated with neutrophil dysfunction, leads to a reduction in phagocytic and microbicidal cell activity, altering virus clearance [8].

In sepsis and systemic inflammatory response syndrome, which are consequences of a severe course of COVID-19, a significant number of immature neutrophil forms appear in the peripheral bloodstream. These immature forms exhibit low functional activity and a pro-inflammatory phenotype, having a longer lifespan and resistance to spontaneous apoptosis [9].

Pregnancy, being a unique physiological state for women, is associated with changes in the immune system. It has been shown that these changes enhance the susceptibility of pregnant women to infections, including SARS-CoV-2 infection [10]. According to Chen G. et al., neutrophilia is characteristic of pregnancy, regardless of whether the pregnant woman is infected with SARS-CoV-2 or not [11]. However, Guleroglu F.Y. et al. claim that the levels of polymorphonuclear leukocytes and neutrophilic granulocytes in the blood are higher in uninfected SARS-CoV-2 pregnant women than in those with COVID-19 [12]. It is also noted that during pregnancy, neutrophilic granulocytes exhibit increased activity and produce reactive oxygen species, which defines the pro-inflammatory nature of immune responses [13]. Lampe et al. observed a significant decrease in the phagocytic index of neutrophilic granulocytes in both healthy and pregnant individuals with pathological pregnancies, which may be relevant to increased infection susceptibility [14].

However, it remains unclear how the functional properties of neutrophilic granulocytes change and how this is related to the development of lung damage in pregnant women with COVID-19. There is also no consensus on the use of clinical blood parameters describing changes in the leukocyte formula in predicting pneumonia development in pregnant women with COVID-19.

Therefore, we believe that investigating polymorphonuclear leukocytes and neutrophilic granulocytes in the blood of pregnant women with COVID-19 is of clinical importance, as cell dysfunction inevitably leads to severe lung damage. The results presented may be of particular interest to studies on the pathogenesis of SARS-CoV-2 infection during pregnancy, and can be used to develop more effective approaches for the diagnosis and prognosis of pneumonia severity in pregnant women with COVID-19.

Additionally, this can be used in the development of more effective approaches to the diagnosis and prognosis of pneumonia severity in pregnant women with COVID-19, which is essential for devising therapeutic measures aimed at minimizing the adverse consequences of the disease for the mother and the fetus.

## 2. Materials and Methods

### 2.1. Study Conditions and Design

A single-moment cross-sectional comparative study was conducted from January to December 2022 at the pulmonology departments of the Amur Regional Clinical Hospital and the Blagoveshchensk City Clinical Hospital. The main group included 54 women in the third trimester of pregnancy with moderate COVID-19 severity. The median age of the participants was 31.0 (29.0; 34.0) years. The sample was formed according to inclusion and exclusion criteria.

The inclusion criteria were as follows: the third trimester of pregnancy; a singleton spontaneous pregnancy; the presence of SARS-CoV-2 antigen in nasopharyngeal and oropharyngeal swabs; clinical symptoms of a respiratory illness; signs of viral pneumonia based on computed tomography (CT) data against a typical clinical picture (and relevant epidemiological history); and informed consent for participation.

Exclusion criteria were as follows: a gestational age less than 28 weeks at the onset of COVID-19; under 18 years of age; multiple pregnancies; cardiovascular diseases, including congenital ones; exacerbation of chronic non-infectious diseases; chronic non-specific lung diseases; extrapulmonary foci of infections; specific bronchopulmonary system diseases; abnormalities in the development of genital organs; sexually transmitted infections; hormonal support with gestagens; smoking; and refusing to participate in the study.

The diagnosis of COVID-19 was made based on the data from laboratory studies for the presence of SARS-CoV-2 RNA using real-time polymerase chain reaction. Computed tomography for pregnant women in the main group was conducted in accordance with the ‘Methodological Recommendations for the Organization of Medical Care for Pregnant Women, Parturients, Women in Labor, and Newborns in the context of the Novel Coronavirus Infection (COVID-19)’ (version 5, 28 December 2021) [15], strictly by the decision of the medical commission. The purpose of conducting computed tomography in pregnant women was the suspicion of a risk of disease progression. The CT of chest organs was performed using programs to limit radiation exposure and protect radiosensitive organs (uterus) and the fetus, applying standard protective measures.

Further subdivision of pregnant women in the main group was conducted based on the extent of lung changes according to CT data. Subgroup 1 (*n* = 43) included pregnant women with minimal lung changes (less than 25%, CT-1), and subgroup 2 included those with moderate lung changes (25–50%, CT-2).

The control group comprised 35 uninfected SARS-CoV-2 women with uncomplicated pregnancies. The median age of the participants was 30.0 (28.0; 35.0) years. Inclusion criteria for the control group were: the third trimester of pregnancy; a singleton spontaneous pregnancy; an absence of respiratory infections during the current pregnancy; and informed consent for participation.

The entire study was conducted in strict accordance with the Helsinki Declaration of the World Medical Association and the rules of clinical practice in the Russian Federation. The study was approved by the Biomedical Ethics Committee of the Federal State Budgetary Scientific Institution “Far Eastern Scientific Center of Physiology and Pathology of Respiration” (protocol No. 140, 10 September 2023). Voluntary informed consent for participation in the study was obtained from each patient.

### 2.2. Blood Samples and Preparation of Specimens

Blood samples from the examined women were taken within the first 24 h of hospitalization using venipuncture with vacuum blood collection systems from the cubital access with tubes containing ethylenediaminetetraacetic acid (EDTA) (Guangzhou Improve Medical Instruments Co., Ltd., Guangzhou, China).

The preliminary preparation of blood for obtaining leukocyte concentrates was performed by centrifugation for 20 min at 1000× *g*. Smears of polymorphonuclear leukocytes were prepared from the obtained pellet in a monolayer fashion using a microcentrifuge for “DIFF-SPIN-2” slides (Statspin, Norwood, MA, USA). The prepared smears were used for cytological examinations.

### 2.3. Cytochemical Research Methods

The determination of succinate dehydrogenase was conducted according to a previously described methodology [16]. Freshly prepared blood smears fixed in alcohol-formalin were incubated in a phosphate buffer (pH 7.2–7.4) containing thiazole blue, trilon B, sodium succinate, and sucrose. The activity of succinate dehydrogenase was assessed by the number of dark blue granules in the cytoplasm of neutrophilic granulocytes.

For the determination of cationic proteins, a previously described method was applied [17]. Prepared blood smears were fixed by incubation in a buffered alcoholic solution of strong green (pH 8.0–8.2), followed by counterstaining the nuclei with a 0.1% aqueous solution of neutral red. The content of cationic proteins was judged by the number of dark blue-brown granules in the cytoplasm of neutrophilic granulocytes, with the nuclei counterstained in red.

The determination of myeloperoxidase was performed using the ready-made kit “DIAKHIM-CYTOSTEIN-MPO” (Saint-Petersburg, Russia). The method is based on the oxidation of benzidine by hydrogen peroxide to form brown oxybenzidine. The activity of myeloperoxidase was assessed by the number of dark brown granules in the cytoplasm of neutrophilic granulocytes, with the nuclei counterstained in red using the Romanovsky–Giemsa stain. The methodology was strictly followed in accordance with the manufacturer’s recommendations.

### 2.4. Microscopy and Evaluation of Cytochemical Reaction Results

Preparations were examined under an immersion system on a “MEIJJI” microscope (Chikumazawa, Japan).

The intensity of staining in all cytological reactions and the number of positive cells were semi-quantitatively evaluated for 100 neutrophils (NEU) by calculating the mean cytological index (MCI) using the previously described method [18]:

MCI = (3 × C + 2 × B + A)/100

where

A is the number of NEU with a weakly positive reaction,
B is the number of NEU with a moderately positive reaction,
C is the number of NEU with a strongly positive reaction.

### 2.5. Neutrophil Extracellular Trap (NET) Investigation

For the study of neutrophil extracellular traps (NETs), smears were fixed in a methylene blue-eosin fixative according to May–Grunwald, followed by staining with Romanovsky–Giemsa azur-eosin. The smears were used to count the number of native undestroyed leukocytes (neutrophils, eosinophils, basophils) and the level of NETs, totaling 200–250 structures. The quantitative counting of NETs was performed using the formula:

NETs, % = *n* NETs/(*n* nat.neutrophils + *n* eosinophils + *n* basophils) × 100%

where *n* NETs—is the number of neutrophil extracellular traps, *n* neutrophils—is the number of native neutrophils, *n* eosinophils—is the number of eosinophils, and *n* basophils—is the number of basophils.

Microscopy of stained smears was performed using the MEKOS-C2 automated microscopy system (Medical Computer Systems LLC, Moscow, Russia) according to the previously described methodology [19].

### 2.6. Investigation of the Number of Polymorphonuclear Leukocytes and the Ratio of Morphological Forms of Neutrophil Granulocytes

Indicators of the number of polymorphonuclear leukocytes and the ratio of morphological forms of neutrophil granulocytes were extracted from the medical records of pregnant women receiving medical care in inpatient settings (form No. 096/1u-20) and individual medical records of pregnant and postpartum women (form No. 111/u-20).

### 2.7. Statistical Data Analysis

Statistical analysis and data processing were performed using the IBM SPSS Statistics software, version 23.0 (Armonk, NY, USA). Quantitative data sets were described using median values (Me), lower and upper quartiles (Q25; Q75). Since most groups had features with distributions that were different from normal, the Mann–Whitney U test was used to test statistical hypotheses when comparing numerical data between two unrelated groups. When comparing multiple samples of quantitative data with distributions different from normal, the Kruskal–Wallis test was applied. Logistic regression analysis and ROC curve analysis were used to assess the diagnostic significance of quantitative indicators in predicting a specific outcome. All differences were considered significant at (*p* < 0.05).

## 3. Results

### 3.1. Clinical Characteristics of Pregnant Groups

Table 1 presents the clinical characteristics of the main and control groups.

As seen in Table 1, the groups were comparable in terms of age (*p* = 0.930), gestational age (*p* = 0.629), and body mass index (BMI) (*p* = 0.785).

According to the radiological computed tomography data in the pregnant women of the main group, the extent of lung involvement in subgroup 1 corresponded to minimal changes, and in subgroup 2—to moderate changes. All of the examined pregnant women in the main group had bilateral lung involvement, with predominant involvement of the right lung detected in 4 (9.3%) cases in subgroup 1, and left-sided localization in 3 (6.98%) cases. Symmetrical damage to both lungs was observed in 36 (83.72%) cases in subgroup 1 and in all 11 (100%) cases in subgroup 2.

The average duration of the disease from the onset of the first symptoms to hospital admission did not differ in the studied subgroups and was 4.0 (2.0; 6.0) days (*p* = 0.130).

The nature of complaints made by pregnant women in the studied subgroups was generally similar (Table 2). From Table 2, it can be seen that in both studied subgroups, the most common symptoms were fever, weakness, and dry cough, followed by a sense of shortness of breath. The frequency of detection and the severity of individual complaints differed between subgroups. In addition, complaints about elevated body temperature and a dry cough were more common in pregnant women from subgroup 2. The severity of the temperature response was also higher in pregnant women in subgroup 2 compared to subgroup 1: 38.0 (37.7; 38.9) °C versus 37.5 (36.6; 38.0) °C (*p* = 0.019). Fever reaching febrile digits was noted in 9 (81.82%) pregnant women in subgroup 2 compared to 14 (32.55%) pregnant women in subgroup 1 (*p* < 0.001). The absence of smell and taste was equally common in pregnant women in subgroup 1 and subgroup 2 (*p* = 0.568). Complaints of chest pain were rare and were noted in pregnant women from subgroup 2. In addition, the regression of symptoms was more prolonged in pregnant women in subgroup 2. Moreover, the median hospital stay for pregnant women in subgroup 2 was 20 (15; 20) days, compared to 15 (12; 16) days for pregnant women in subgroup 1 (*p* = 0.001).

### 3.2. Characteristics of Polymorphonuclear Leukocytes and Morphological Forms of Neutrophils in the Blood of Pregnant Women with Moderate COVID-19 Severity

**Figure 1:**
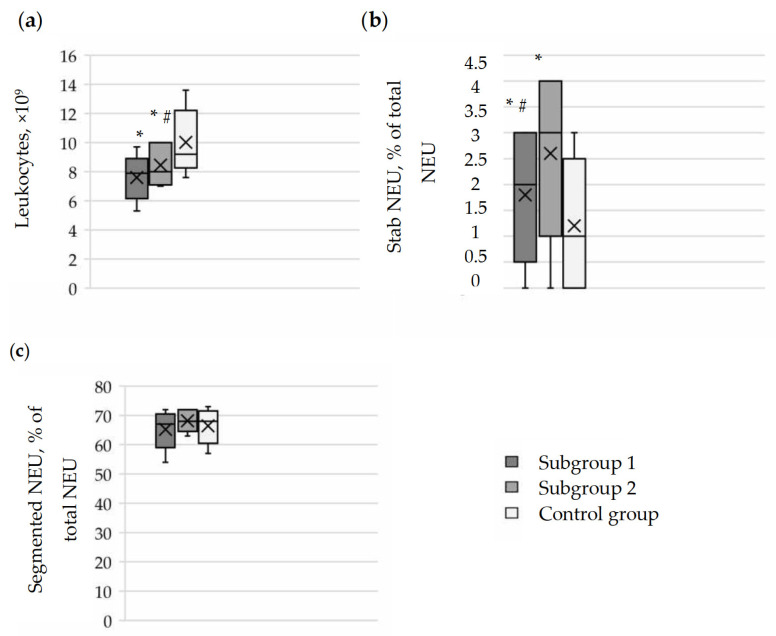
Comparative analysis of the total number of polysegmentonuclear leukocytes and the content of morphological forms of neutrophils in the blood of pregnant women in the subgroups of the main group and in the control group. (**a**) Assessment of the distribution of the total number of leukocytes based on clinical blood analysis in the pregnant women of the study groups. (**b**) Analysis of the percentage content of band neutrophils out of the total number of neutrophils in the blood of the pregnant women of the study groups. (**c**) Analysis of the percentage content of segmented neutrophils out of the total number of neutrophils in the blood of the pregnant women of the study groups. The results are presented as median, lower and upper quartiles with the overall range of value changes. Comparison of data in the groups was conducted using the Kruskal–Wallis test, * *p* < 0.05 compared to the control group, # *p* < 0.05 significance of differences between subgroup 1 and subgroup 2 in the main group.

According to the clinical blood analysis of pregnant women in subgroups 1 and 2, the number of polymorphonuclear leukocytes was 7.9 (7.0; 8.1) (*p* < 0.0001) and 8.0 (7.2; 10.0) (×10^9^) (*p* = 0.003), respectively, which was lower than in the control group (9.2 (8.9; 10.8) (×10^9^)) (Figure 1). Comparison within the main group of pregnant women showed that the number of polymorphonuclear leukocytes in the blood was slightly higher in subgroup 2 than in subgroup 1 (*p* = 0.03).

The analysis of the percentage ratio of morphological forms of neutrophils in the blood of pregnant women showed that the number of band forms in subgroup 1 was higher (2.0 (1.0; 3.0)%) than in the control group (1.0 (0.0; 2.0)%; *p* < 0.0001) and lower than in subgroup 2 (3.0 (2.0; 4.0)%; *p* = 0.002). At the same time, there were no significant differences in the number of segmented neutrophils in the blood of pregnant women in subgroup 1 (67.0 (64.0; 69.0)%; *p* = 0.216) and in subgroup 2 (68.0 (66.0; 72.0)%; *p* = 0.273) compared to the control group (68.0 (64.0; 70.0)%). There were also no differences when comparing similar indicators between subgroups 1 and 2 (*p* = 0.115). It is likely that the left shift of the leukocyte formula in pregnant women in subgroup 1 is a result of the body’s response to infection and inflammation. These changes may contribute to the overall functional and microbiocidal activity of neutrophil granulocytes.

### 3.3. Evaluation of the Functional and Microbiocidal Activity of Neutrophil Granulocytes in the Blood of Pregnant Women with Moderate COVID-19

The next stage of our research involved assessing the functional and microbiocidal activity of neutrophil granulocytes in the blood of pregnant women in the study groups. This assessment included performing cytochemical reactions, examining smears under a microscope, and calculating the mean cytochemical index (MCI).

**Figure 2:**
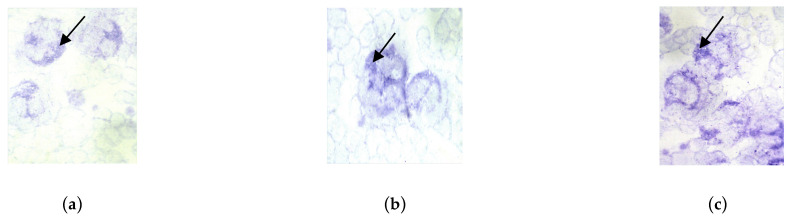
Assessment of the intensity of the cytochemical reaction for succinate dehydrogenase in neutrophil granulocytes by the tetrazolium method. Distribution of reaction products in the cytoplasm in the form of blue-violet granules, indicated by arrows. Magnification 100×, immersion, light microscopy. Intensity of staining of reaction products for succinate dehydrogenase: (**a**) Subgroup 2; (**b**) Subgroup 1; (**c**) Control group.

**Figure 3:**
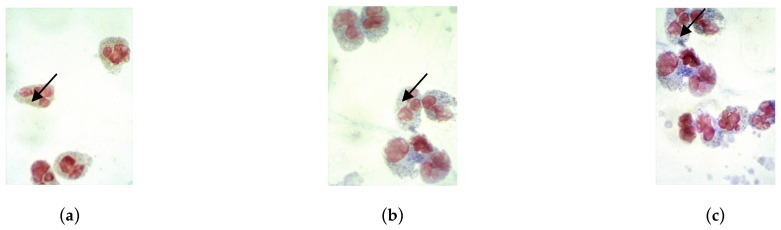
Assessment of the intensity of the cytochemical reaction to cationic proteins in neutrophil granulocytes. Distribution of reaction products in the cytoplasm (in the form of blue-brown granules) are indicated by arrows. Magnification 100×, immersion, light microscopy. Intensity of staining of reaction products for cationic proteins: (**a**) Subgroup 2; (**b**) Subgroup 1; (**c**) Control group.

**Figure 4:**
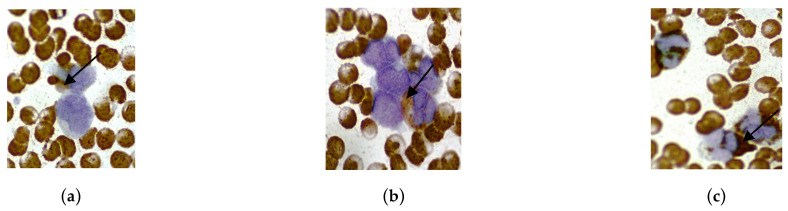
Assessment of the intensity of the cytochemical reaction for myeloperoxidase in neutrophil granulocytes using the benzidine method. Distribution of reaction products in the cytoplasm (in the form of brown granules) are indicated by arrows. Magnification 100×, immersion, light microscopy. Intensity of staining of reaction products for cationic proteins: (**a**) Subgroup 2; (**b**) Subgroup 1; (**c**) Control group.

Microphotographs of cytochemical reactions for mitochondrial succinate dehydrogenase and granular antimicrobial peptides (lysosomal cationic proteins, myeloperoxidase) in neutrophil granulocytes are presented in Figure 2, Figure 3 and Figure 4. Microscopic examination of smears revealed a diffuse distribution of reaction products in the cytoplasm of neutrophils, in the form of granules of varying intensity of staining. The highest intensity of staining for peptide reaction products was observed in pregnant women in the control group, whereas the lowest was in subgroup 2 and, correspondingly, in subgroup 1.

**Figure 5:**
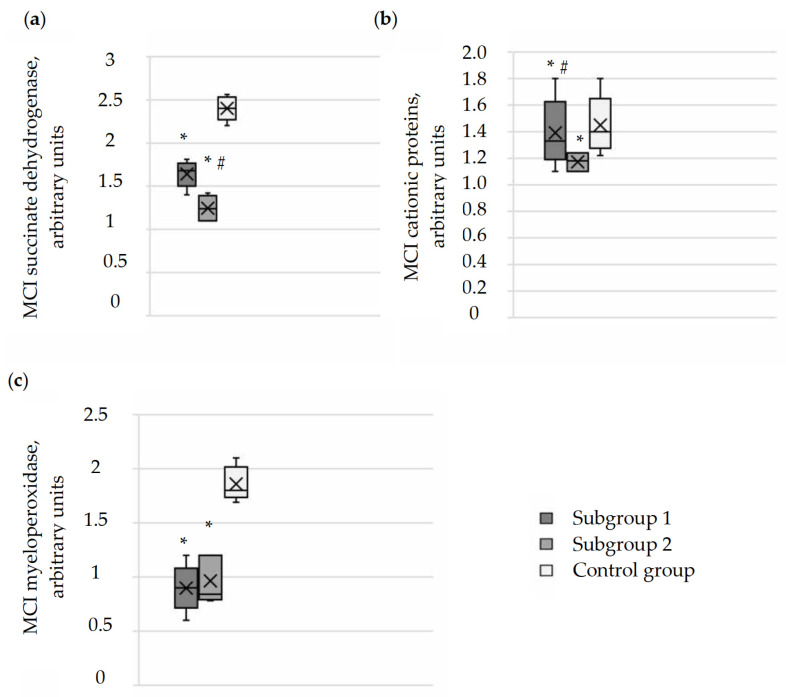
Assessment of the functional activity of neutrophil granulocytes based on the average cytochemical coefficient of succinate dehydrogenase, cationic proteins, and myeloperoxidase in the subgroups of pregnant women in the main group and in the control group. (**a**) Comparative analysis of succinate dehydrogenase indicators in neutrophils of blood in the pregnant study groups. (**b**) Assessment of the amount of cationic proteins in neutrophils of blood in the pregnant study groups. (**c**) Comparative analysis of myeloperoxidase indicators in neutrophils of blood in the pregnant study groups. The results are presented as median, lower and upper quartiles with the overall range of value changes. Comparing the data groups was conducted using the Kruskal–Wallis test, * *p* < 0.05 compared to the control group, # *p* < 0.05 significance of differences between subgroup 1 and subgroup 2 in the main group.

The calculation of the MCI confirmed that the level of succinate dehydrogenase in neutrophil granulocytes was reduced in pregnant women in subgroups 1 (1.68 (1.6; 1.72); *p* < 0.0001) and 2 (1.24 (1.1; 1.36); *p* < 0.0001) compared to the control group (2.4 (2.34; 2.5)) (Figure 5). Interestingly, the lowest MCI values were observed in pregnant women in subgroup 2, which could indicate the development of mitochondrial dysfunction and energy imbalance in response to the impairment of lung ventilation function during SARS-CoV-2 damage and oxygen deficiency in the blood. Hypoxemia and hypoxia in the main group of pregnant women may act as a factor initiating oxidative stress reactions and disrupting the microbicidal properties of cells, which are determined by the state of granular peptides such as cationic proteins and myeloperoxidase.

When calculating the MCI, it was found that the values of cationic proteins in neutrophil granulocytes in pregnant women in subgroup 1 (1.33 (1.28; 1.45); *p* < 0.0001) were higher than in subgroup 2 (1.18 (1.1; 1.24); *p* < 0.0001) and lower than in the control group (1.4 (1.33; 1.5)). The MCI values of myeloperoxidase were also reduced in pregnant women in subgroup 1 (0.9 (0.83; 0.96); *p* < 0.0001) and in subgroup 2 (0.84 (0.78; 1.2); *p* < 0.0001) compared to the control group (1.8 (1.78; 1.93)). When comparing between subgroups 1 and 2, no differences in myeloperoxidase MCI values were found (*p* = 0.587). The lowest MCI values for myeloperoxidase in neutrophil granulocytes were observed in pregnant women in subgroup 2.

Based on these results, it can be concluded that the decrease in the amount of myeloperoxidase in neutrophil granulocytes in the main group of pregnant women could be due to its increased consumption during phagocytosis or release into the systemic bloodstream. A similar conclusion can be drawn regarding cationic proteins. The reduction of their quantity in the lysosomes of neutrophil granulocytes may also be associated with active release from cells due to the destabilization of the cell membrane by SARS-CoV-2. However, these data require further confirmation.

**Figure 6:**
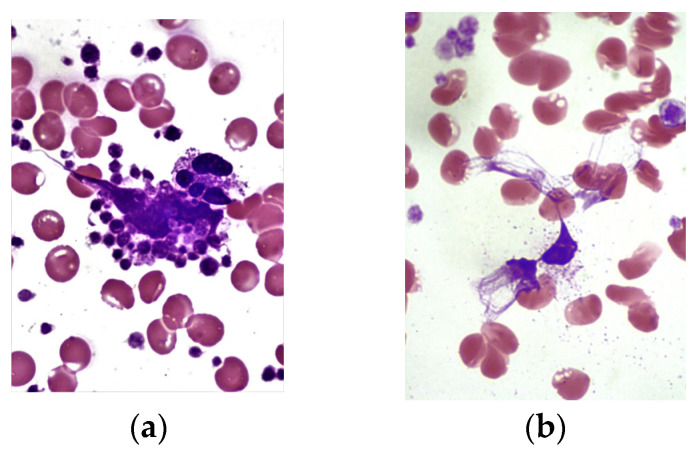
Evaluation of the content of neutrophil extracellular traps (NETs) in the blood of pregnant women in the main group using the Romanowsky-Giemsa method. Magnification 100×, immersion, light microscopy. (**a**) Presence of platelet-neutrophil complexes and NETs in pregnant women in subgroup 2; (**b**) Decondensed DNA of neutrophils in complex with granulated proteins in the extracellular space in pregnant women in subgroup 1.

In relation to peculiarities of neutrophilic granulocyte functioning in pregnant women in the main group, we also assessed the state of NETs formation and the removal of NETosis products, which can be considered as pathological markers of inflammation and vascular impairment. Some variants of NETs formations in pregnant women in subgroups 1 and 2 are presented in Figure 6. Microscopic examination of blood smears in pregnant women in subgroup 2 often revealed platelet-neutrophil complexes and NETs. In pregnant women in subgroup 1, a common sign of neutrophil death was the presence of a dense DNA network with granulated proteins in the extracellular space.

**Figure 7:**
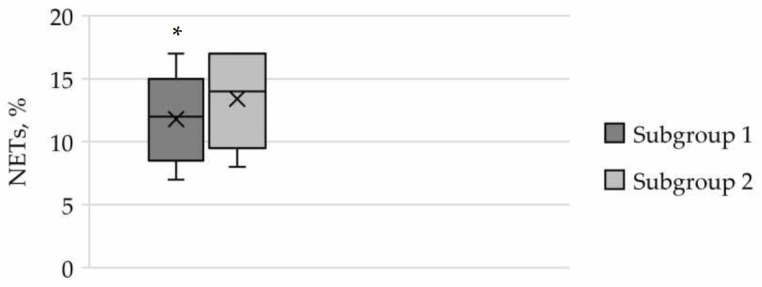
Evaluation of the number of neutrophil extracellular traps (NETs) as a proportion of the total number of leukocytes in the blood of pregnant women in the subgroups of the main group. The results are presented as median, lower and upper quartiles with the overall range of value changes. The comparison of data in the subgroups was conducted using the Mann–Whitney U test, * *p* < 0.05.

In the quantification of NETs, a significant increase was observed in pregnant women in subgroup 2 (14.0 (11.0; 17.0)%) compared to subgroup 1 (12.0 (10.0; 13.0)%; *p* = 0.002) (Figure 7).

The results obtained by us confirm the involvement of neutrophilic granulocytes in the severity of lung damage in pregnant women in the main group.

### 3.4. Predictors of Pneumonia Severity in Pregnant Women with COVID-19

For the search of predictors of pneumonia severity in pregnant women in the main group, a multivariate regression analysis was conducted, incorporating clinical (age, BMI, gestational age, lung damage characteristics from computer tomography), laboratory (polymorphonuclear leukocytes, rod-shaped and segmented neutrophils), and cytochemical (succinate dehydrogenase, myeloperoxidase, cationic proteins, NETs) parameters as independent variables. The dependent variable was the degree of lung involvement based on computed tomography data.

The logistic regression method indicated that succinate dehydrogenase (OR = 0.75, 95% CI: 0.6–0.9, *p* = 0.002) and NETs (OR = 0.76, 95% CI: 0.64–0.91, *p* = 0.003) could be utilized as independent predictors of pneumonia severity in pregnant women in the main group. Other independent variables, both clinical (age, BMI, gestational age, lung damage characteristics from computer tomography) and laboratory (myeloperoxidase, cationic proteins), did not exert a significant influence on the severity of lung damage in pregnant women (*p* > 0.05).

To check the consistency of the model with the original data, the Hosmer-Lemeshow goodness-of-fit test was used: classification—goodness-of-fit test, χ^2^ = 5.14; degrees of freedom—8; and *p* = 0.743. The test indicates the level of significance at which the hypothesis of acceptably minor discrepancies between the actual and model classification, reflecting the severity of lung damage and development of pneumonia in pregnant women with COVID-19, is not rejected. In this case, for the presented model, the achieved significance level of *p* > 0.05 indicates the model’s consistency with real data.

Thus, a reduction in the level of succinate dehydrogenase in neutrophil granulocytes concurrently with an increase in NETs in the blood enhances the likelihood of severe lung damage and the development of pneumonia in pregnant women with COVID-19.

**Figure 8:**
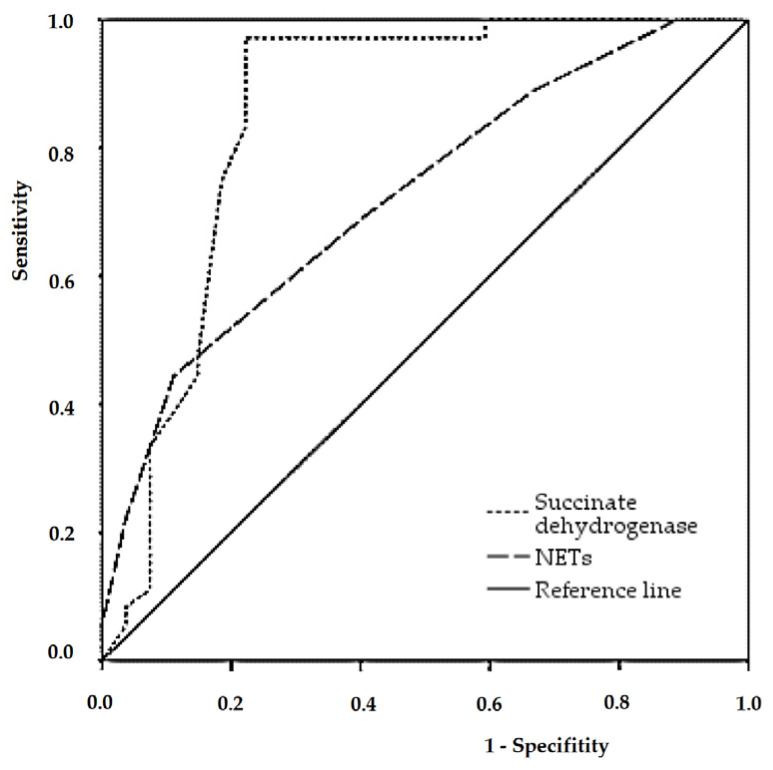
ROC curves for predictors of pneumonia severity in pregnant women with COVID-19.

Subsequent ROC analysis confirmed the feasibility of using succinate dehydrogenase and NETs as predictors of pneumonia severity in pregnant women with COVID-19 (Table 3, Figure 8).

The diagnostic efficiency of each, calculated based on their sensitivity and specificity, respectively, was 69.7% for NETs and 81% for succinate dehydrogenase. Each of these parameters was characterized by favorable prognostic ability, as evidenced by the values of the area under the curve (AUC). The optimal threshold probability value for succinate dehydrogenase was equal to or less than 1.43, and for NETs—equal to or greater than 11.5%.

**Figure 9:**
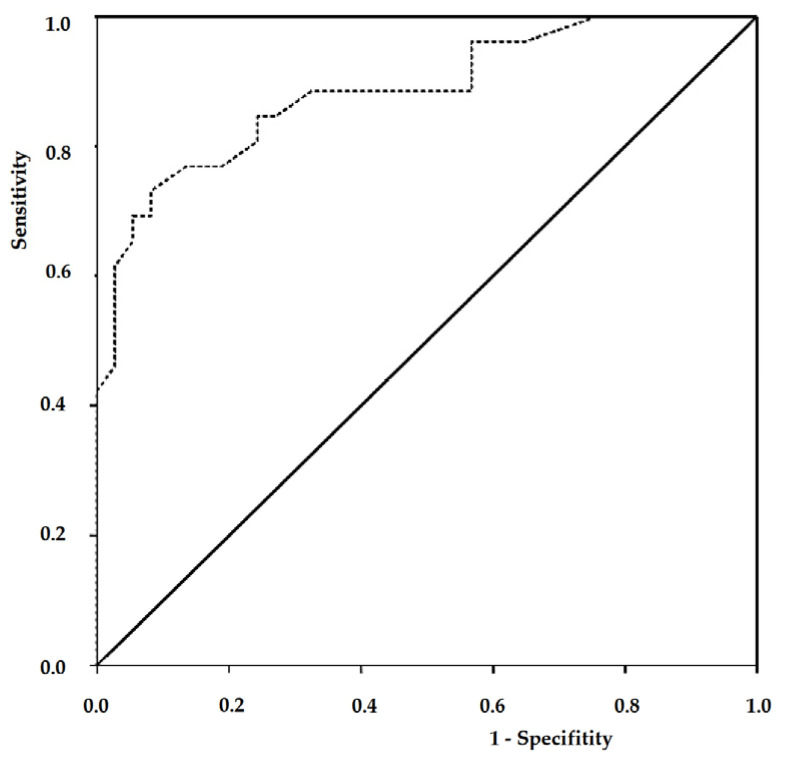
ROC curve for assessing the effectiveness of the final logistic regression model.

Subsequently, an evaluation of the effectiveness of the created prognostic model was conducted using two functions (Table 4, Figure 9).

The area calculated under the ROC curve corresponded to a model of very high quality. The optimal threshold probability value for sensitivity and specificity of this model was 0.264. According to the validation results, the diagnostic efficiency of the model was at 84.1%, indicating its potential use in predicting the severity of pneumonia in pregnant women with COVID-19.

## 4. Discussion

It is known that the structural and metabolic status of polymorphonuclear leukocytes is closely linked to the performance of their physiological functions and is determined by activation, adhesion, cell chemotaxis, antigen uptake, killing, and breakdown. We observed an increase in the total leukocyte count in the blood of pregnant women with COVID-19, with a mean lung volume change relative to pregnant women with minimal lung volume changes based on computed tomography data.

The observed left shift in the leukocyte formula and the increased circulation of immature band forms of neutrophils in the blood also indicate the severity of the pathological process due to impaired immune reactivity in pregnant women with COVID-19. This brings into question the advantages of using glucocorticosteroids as regulators of neutrophilic inflammation in pregnant women with COVID-19 compared to cases in which their use is justified by the risk of preterm birth [20]. In this respect, the study by Sinha S. et al. is of great importance, showing that dexamethasone affects the polarization of neutrophils and the development of the innate immune response, as opposed to the proliferation of their immature forms [21]. It is quite possible that for a reasoned decision before prescribing glucocorticosteroids to pregnant women with COVID-19, it is necessary to take into account the clinical status of the mother and the gestational age of the fetus [22,23], in addition to paying attention to the rate of penetration of glucocorticosteroids through the placenta, to reduce the risks of fetal growth retardation and the birth of newborns with low body weight [24].

Several studies have shown that the accompanying COVID-19 inflammation causes destabilization of the cell membrane of neutrophilic granulocytes and their transformation, disrupts adhesive and emigration properties, impedes antigen uptake, and also causes capping [25,26]. Other data indicate that the inflammatory process significantly reduces the activity of key enzymes of anaerobic oxidation and ATP reserves in neutrophils, leading to the formation of secondary mitochondrial dysfunction [27]. We confirmed that in pregnant women with an average volume of lung changes, cytochemical indicators of succinate dehydrogenase significantly decreased, which could indicate a breakdown in the mechanisms of rapid adaptation in response to hypoxia and the accumulation of active forms of oxygen [28]. The same changes were found in the indicators of myeloperoxidase and lysosomal cationic proteins. It is possible that the decrease in myeloperoxidase could be due to an increase in consumption during phagocytosis or release and entry into the systemic bloodstream. A similar conclusion can be made regarding cationic proteins. Their decrease may be related to active ejection from cells as a result of destabilization of the cell membrane by SARS-CoV-2. The identified patterns may indicate the depletion of mechanisms that determine the development of secondary granulocytopenias and the severity of lung damage in pregnant women with COVID-19.

According to Zhang R. et al., functionally altered neutrophils may actively enter NETosis, forming NETs and their demise in patients with COVID-19 [29]. Our studies confirm that in pregnant women with an average volume of lung changes, the number of platelet-neutrophil complexes and NETs significantly increased, which can be considered an objective sign of disruption of alveolar microcirculation and severity of lung damage. The method we used to count NETs in blood smears, compared to other methods [30], has several advantages, including simplicity and general availability, allowing its use in routine clinical practice.

According to multidimensional regression analysis, based on the use of clinical (age, BMI, gestational age, lung damage characteristics based on computed tomography data), laboratory (polymorphonuclear leukocytes, segmented and banded neutrophils), and cytochemical (succinate dehydrogenase, myeloperoxidase, cationic proteins, NETs) parameters as independent variables, only succinate dehydrogenase and NETs demonstrated favorable prognostic capabilities. The calculated regression model for assessing the severity of pneumonia in pregnant women with COVID-19 demonstrated high sensitivity, specificity, and diagnostic effectiveness. No statistically significant differences were found in the quantitative indicators of succinate dehydrogenase and NETs between pregnant women with COVID-19 with minimal and moderate lung changes, which also confirms the high quality of the model and its potential use in predicting the severity of pneumonia in pregnant women with COVID-19.

In our study, there are limitations in terms of sample validation, and its enlargement is necessary to confirm the accuracy of the prognostic model. Furthermore, the mechanisms of neutrophil mitochondrial dysfunction and its impact on the NETotic transformation of cells, which support inflammation and lung tissue damage in COVID-19, require further confirmation.

## 5. Conclusions

The results of this study provide compelling evidence that COVID-19 in pregnant women leads to an increase in polymorphonuclear leukocytes and band forms of neutrophils in the blood, dependent on the severity of lung damage. There is also a decrease in cytochemical indicators of succinate dehydrogenase, myeloperoxidase, and cationic proteins in neutrophil granulocytes, and an increase in platelet-neutrophil complexes and NETs. The application of logistic regression allowed for the calculation of independent predictors of pneumonia severity in pregnant women with COVID-19—neutrophil succinate dehydrogenase and NETs. Their combined use increases the diagnostic effectiveness of the model in assessing the severity of pneumonia in pregnant women with COVID-19.

## Figures and Tables

**Table 1:** Clinical Characteristics of the Investigated Pregnant Groups.

Groups	Main Group	Control Group	*p*-Value
**Group size**	54	35	
**Age, years**	31.0 (29.0; 34.0)	30.0 (28.0; 35.0)	0.930
**Gestational Age, weeks**	37.0 (35.0; 38.0)	37.0 (35.0; 38.0)	0.629
**BMI (Body Mass Index)**	26.9 (24.3; 31.2)	25.0 (24.1; 26.4)	0.785

Note: *p*, significance of differences between the main group of pregnant women with control the group of pregnant women.

**Table 2:** Frequency of Occurrence of Clinical Symptoms in the Investigated Subgroups of Pregnant Women.

Groups	Subgroup 1		Subgroup 2		*p*-Value
Group Size	43		11		
	Absolute No.	%	Absolute No.	%	
**Fever**	19	44.19	11	100	<0.001
**Weakness**	24	55.81	7	63.64	0.313
**Dry cough**	27	62.79	11	100	<0.001
**Sore throat**	11	25.58	0	0	-
**Chest pain**	0	0	3	27.27	-
**Loss of smell, taste**	17	39.53	5	45.45	0.568
**Shortness of breath**	34	62.79	7	63.64	0.884

Note: *p*, significance of differences between subgroups of pregnant women in the main group.

**Table 3:** Characteristics of ROC curves for predictors of pneumonia development in pregnant women with COVID-19.

Predictors	Succinate Dehydrogenase, Arbitrary Units	NETs, %
**Sensitivity, %**	91.7	69.4
**Specificity, %**	77.8	60
**Area under the curve**	0.853	0.725
**Standard error**	0.057	0.063
**95% Confidence Interval**	0.741–0.964	0.601–0.849
***p*-value (against AUC = 5)**	<0.0001	0.002

Note: *p* (against AUC = 0.5)—the level of significance at which the evaluated AUC is statistically significantly different from the non-informative value of 0.5.

**Table 4:** Characteristics of the ROC curve for the logistic regression model assessing the development of pneumonia in pregnant women with COVID-19.

Characteristics	
**Sensitivity, %**	84.6
**Specificity, %**	75.7
**Area under the curve**	0.885
**Standard error**	0.044
**95% CI**	0.798–0.971
***p*** **-value (against AUC = 0.5)**	<0.0001

Note: *p* (against AUC = 0.5)—the level of significance at which the evaluated AUC is statistically significantly different from the non-informative value of 0.5.

## Data Availability

The data presented in this study are available on request from the corresponding author.
